# Unexpectedly high *Plasmodium* sporozoite rate associated with low human blood index in *Anopheles coluzzii* from a LLIN-protected village in Burkina Faso

**DOI:** 10.1038/s41598-018-31117-x

**Published:** 2018-08-24

**Authors:** Marco Pombi, Maria Calzetta, Wamdaogo M. Guelbeogo, Mattia Manica, Eleonora Perugini, Verena Pichler, Emiliano Mancini, N’Fale Sagnon, Hilary Ranson, Alessandra della Torre

**Affiliations:** 1 Dipartimento di Sanità Pubblica e Malattie Infettive, Laboratory affiliated to Istituto Pasteur Italia - Fondazione Cenci Bolognetti, Sapienza Università di Roma, Rome, 00185 Italy; 2 Centre National de Recherche et Formation sur le Paludisme (CNRFP), Ouagadougou, 01 BP 2208 Burkina Faso; 3 Dipartimento di Biodiversità ed Ecologia Molecolare, Centro Ricerca e Innovazione, Fondazione Edmund Mach, via E. Mach 1, 38010 San Michele all’Adige, Italy; 4 Università di “Roma Tre”, Dipartimento di Scienze, Rome, 00154 Italy; 5 Liverpool School of Tropical Medicine, Department of Vector Biology, Liverpool, L3 5QA UK

## Abstract

Despite the effectiveness of mass distribution of long-lasting insecticidal nets (LLINs) in reducing malaria transmission in Africa, in hyperendemic areas such as Burkina Faso the burden of malaria remains high. We here report the results of a 4-month survey on the feeding habits and *Plasmodium* infection in malaria vectors from a village in Burkina Faso one year following a national LLIN distribution programme. Low values of human blood index (HBI) observed in the major malaria vectors in the area (*Anopheles coluzzii*: N = 263, 20.1%; *An. arabiensis*: 5.8%, N = 103) are consistent with the hypothesis that LLINs reduced the availability of human hosts to mosquitoes. A regression meta-analysis of data from a systematic review of published studies reporting HBI and sporozoite rates (SR) for *An. gambiae* complex revealed that the observed SR values (*An. coluzzii*: 7.6%, N = 503; *An. arabiensis*: 5.3%, N = 225) are out of the ranges expected based on the low HBI observed. We hypothesize that a small fraction of inhabitants unprotected by bednets acts as a “core group” repeatedly exposed to mosquito bites, representing the major *Plasmodium* reservoir for the vectors, able to maintain a high risk of transmission even in a village protected by LLINs.

## Introduction

**Figure 1:**
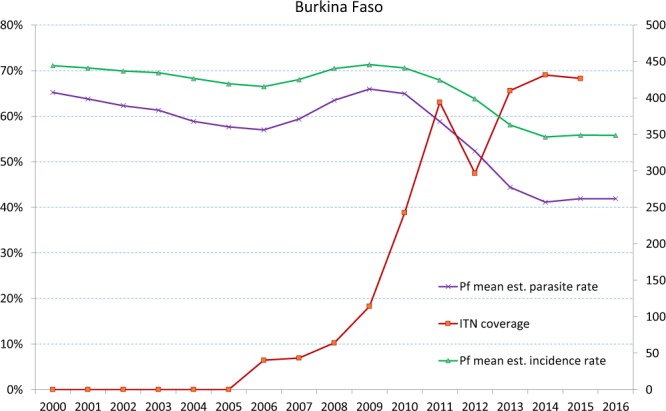
Annual countrywide data on malaria cases for Burkina Faso, estimated per 1,000 inhabitants (2–10 year olds). Left vertical axis: percentage of the population protected by Insecticide Treated Nets (ITN), in red; estimated prevalence of *Plasmodium falciparum* (*Pf*) parasite rate in 2–10 year olds, in purple. Right vertical axis: *Plasmodium falciparum* (*Pf*) estimated incidence per 1,000 inhabitants, in green (source: Malaria Atlas Project, www.map.ox.ac.uk).

Malaria incidence and mortality have globally decreased in the last decade thanks to implementation of integrated control measures, including effective anti-malarial treatments, better health infrastructures, the widespread application of measures reducing human-vector contact (i.e. insecticide treated nets (ITNs), long-lasting insecticide treated nets (LLINs), and indoor residual spraying, IRS)^1–4^. It is estimated that 68% of the 663 million malaria cases prevented in Africa since the year 2000 are due to the usage of LLINs^4^. Between 2008 and 2010, 254 million ITNs were supplied to countries in sub-Saharan Africa, bringing the proportion of African households in possession of a net increased from 3% in 2000 to 50% in 2010^5^. Yet, most of sub-Saharan Africa continues to carry a disproportionately high share of the global malaria burden of malaria deaths^6–10^. This is certainly the case for Burkina Faso where raising of LLIN coverage from 20% to 70% between 2009 and 2014 led to a significant reduction of malaria prevalence, but did not significantly affect malaria annual incidence^1,4,11–19^ (Fig. 1). Low levels of bednet usage^20,21^ and/or reduced efficacy in preventing vector/human contact due to physiological and behavioural resistance of mosquito vectors to pyrethroids used to impregnate the nets^2,22–25^ may have concurred in producing this heterogeneous efficacy.

Entomological studies represent an important component for the full understanding of the impact of LLINs on malaria incidence and transmission dynamics. In Burkina Faso, four major vectors are responsible of most malaria burden, i.e. *Anopheles coluzzii*, *Anopheles gambiae sensu stricto*, *Anopheles arabiensis*, and *Anopheles funestus*. Their ecological features, genetic heterogeneity and plastic behaviours, together with their complementary geographic/seasonal distribution, are major threats to an effective control of malaria in the country^23,26–38^. In fact, these anthropophilic and endophilic species have demonstrated the ability to easily switch to more zoophilic and/or exophilic behaviours as a consequence of the implementation of vector control measures^39–41^.

**Figure 2:**
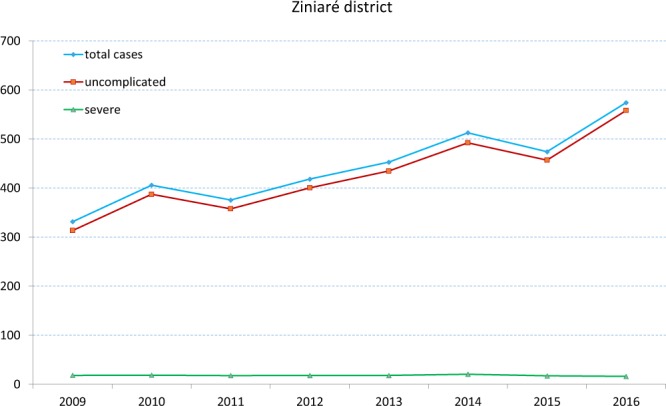
Annual data on malaria cases (blue = total, red = uncomplicated, and green = severe) from 2009 to 2016, calculated per 1,000 inhabitants, in the district of Ziniaré, Burkina Faso. Source: Ministry of Health of Burkina Faso (http://www.cns.bf/spip.php?id_rubrique=17&page=publdetails).

We here present the result of an entomological survey carried out the year after a LLIN mass-distribution campaign in a village of Ziniaré health district in Burkina Faso where high levels of malaria incidence were observed^11–14,19^ (Fig. 2). In order to assess whether the ratio between *Plasmodium* sporozoite rate (SR) and human blood index (HBI) in the study village was in line with those observed in other Afrotropical villages with high LLIN coverage, we compared it with the estimated ratios in Afrotropical villages with different LLIN coverage, as obtained by a systematic review and meta-analysis of published data indexed in PubMed database. Results suggest that, whilst LLIN use led to a strong reduction in human-vector-contact, high *Plasmodium* infection rates were still present in the mosquito vector population from the study village, suggesting a residual high risk of infection for unprotected inhabitants both indoors and outdoors, despite the high LLIN coverage.

## Results and Discussion

### *Plasmodium* infection rates

Overall, *Plasmodium* infection rates obtained by nested-PCR on DNA-templates extracted from the entire carcasses of 1,115 *An. gambiae* s.l. female mosquitoes (hereafter, whole mosquito infection, WMI) were 11.2% for *An. coluzzii*, 9.4% for *An. arabiensis* and 12.5% for *An. gambiae s.s*. (Table 1). PCR-bands amplified from 10 *Plasmodium*-positive specimens were sequenced and identified as *Plasmodium falciparum*-specific.

**Table 1:** *Plasmodium* infection rates in *Anopheles gambiae* s.l. females (entire carcasses) collected in the village of Goden (Burkina Faso) during 2011 rainy season either indoor (IN) or outdoor (OUT).

Species	Position	August	September	October	November	Total
*An. coluzzii*	IN	11.0%	11.5%	13.5%	10.8%	11.6%
	OUT	11.9%	8.7%	13.2%	10.3%	10.7%
	**Total**	**218**	**204**	**157**	**197**	**776**
*An. arabiensis*	IN	9.4%	15.4%	6.3%	10.0%	9.9%
	OUT	13.2%	4.3%	6.5%	22.6%	9.1%
	**Total**	**70**	**106**	**62**	**61**	**299**
*An. gambiae* s.s.	IN	0%	50.0%	50.0%	20.0%	36.4%
	OUT	0%	5.6%	0%	0%	3.4%
	**Total**	**5**	**20**	**9**	**6**	**40**

No significant differences in *Plasmodium* infection rates were observed among species, sampling methods, indoor *vs* outdoor collections, and months of sampling (GLMM-1, Table (a) in Supplementary File 1). The proportion of observed WMI was within the 95% confidence intervals computed by simulating model results (Figure (a) in Supplementary File 1).

Sporozoite Rates (SRs) and Oocyst Rates (ORs) obtained using DNA extracted either from heads + thoraxes or abdomens of 728 females collected by BP and PIT, were 7.6% and 5.2% for *An. coluzzii*, and 5.3% and 4.9% for *An. arabiensis*, respectively (Table 2; rates are not reported for *An. gambiae* due to the small sample size; N = 31). No statistically significant differences were observed between species, sampling methods, indoor vs outdoor collections, and months of sampling (GLMM). *Plasmodium* infection was detected in heads + thoraxes and abdomens of the same mosquito in 8 specimens (1.1%) only.

**Table 2:** *Plasmodium* infection rates in *Anopheles coluzzii* and *An. arabiensis* females collected by active aspiration indoors and outdoors in the village of Goden (Burkina Faso) during the 2011 rainy season.

Species	infection	August	September	October	November	Total
*An. coluzzii*	Sporozoite Rate (SR)	8.8%	5.3%	3.6%	7.3%	6.4%
	Oocyst Rate (OR)	1.5%	4.5%	4.5%	5.6%	4.0%
	SR + OR	1.5%	0.0%	2.7%	0.8%	1.2%
	Negative	88.3%	90.2%	89.1%	86.3%	88.5%
	**Total**	**137**	**133**	**110**	**124**	**503**
*An. arabiensis*	Sporozoite Rate (SR)	7.8%	0.0%	0.0%	15.4%	4.4%
	Oocyst Rate (OR)	2.0%	3.6%	5.9%	5.1%	4.0%
	SR + OR	0.0%	0.0%	0.0%	5.1%	0.9%
	negative	90.2%	96.4%	94.1%	74.4%	90.7%
	**Total**	**51**	**84**	**51**	**39**	**225**

### Blood-meal origin identification

Blood meal origin identification was successful for 63% of the 602 *An. gambiae* s.l. females collected with blood-traces in the abdomens (Table 3). GLM-a analysis showed differences in rates of successful identification between methods of collections (73.3% and 58.7% for the 116 and 303 females collected by SRB and BP-PIT, respectively; likelihood ratio test, df = 3, deviance = 10.43, p-value = 0.015, Supplementary File 2), confirming that that presence of glue in SRB-collected samples does not represent a constraint to molecular analyses^32^. GLM-a also highlighted that the sensitivity of the PCR technique was affected by the amount of blood in the abdomens and/or by its partial digestion, being 74.5% in freshly/fully-fed females (N = 102), and 62.9% and 58.5% in half gravid (N = 35) and partially-fed mosquitoes (N = 282), respectively (Table (b) and Figure (a) and (B) in Supplementary File 2). This is most likely due to a more abundant and/or less degraded DNA template in freshly/fully-fed females, as already reported by Kent & Norris^42^. This is also consistent with the higher blood-meal identification rate in SRB-collected specimens, which included a lower proportion of half-gravid and partially-fed females. Overall, ~25% of good-quality blood-meals (corresponding to the proportion of non-identified blood-meals in fully-fed females, in which an optimal DNA-template can be assumed to have been extracted) were not identified. This not-negligible proportion is in line with literature data on *An. gambiae* complex feeding habits^40,43–46^ and is likely due to blood-meals carried out on non-tested hosts, such as chickens (which were not tested, as *An. coluzzii/An. gambiae* are not known to be ornithophilic)^46^, donkeys (which were rare in the village), or wild birds/mammals.

**Table 3:** Origin of blood-meal in species of the *Anopheles gambiae* complex collected indoors and outdoors in the village of Goden (Burkina Faso) during the 2011 rainy season. UN-ID = unidentified blood-meals.

Species	cow	human	pig	dog	goat	cow/hum	dog/cow	Total identified	UN-ID	Total
*An. coluzzii*	70.3%	19.0%	4.9%	3.4%	1.1%	1.1%	0.0%	263	37.2%	419
*An. arabiensis*	79.6%	5.8%	7.8%	2.9%	2.9%	0.0%	1.0%	103	35.6%	160
*An. gambiae*	78.6%	7.1%	7.1.%	0.0%	7.1%	0.0%	0.0%	14	39.1%	23
**Total**	73.2%	15.0%	5.8%	3.2%	1.8%	0.8%	0.3%	380	36.9%	602

**Figure 3:**
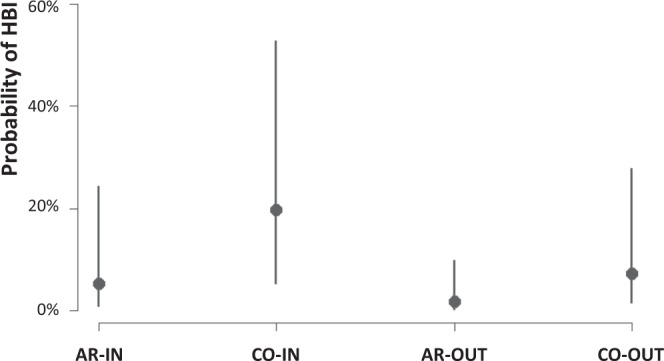
Results of the Binomial GLMM-2 investigating the Human Blood Index in *An. coluzzii* and *An. arabiensis* fed mosquitoes. The dots represent fitted values: vertical bands are the 95% confidence intervals. On the y-axis the probability of a human blood meal (HBI), AR = *An. arabiensis*, CO = *An. coluzzii*. IN = indoor collection, OUT = outdoor collection.

Overall, the Human Blood Index (HBI) calculated only specimens with successful blood-meal identification was significantly higher for *An. coluzzii* (20.1%, including 3 specimens showing a mixed cow-human blood meal) than for *An. arabiensis* (5.8%) and *An. gambiae* s.s. (4.3%), (Table 3; Fig. 3; Table (c) in Supplementary File 2). The higher HBI observed for *An. coluzzii* compared to *An. arabiensis* is expected based on the known higher anthropophily of the former species^38,43,46^, while the low HBI observed in highly anthropophilic *An. gambiae* s.s. may be an artefact of the small sample size.

Due to the expected presence of unidentified blood-meals in mosquitoes feeding on animals different from the tested ones, the reported HBI values likely represent an overestimate of the real HBI in the village. In fact, when calculated on the overall fully-fed female sample (including specimens with non-identified blood-meals), HBIs were 12.6% and 3.8% in *An. coluzzii* and *An. arabiensis*, respectively. HBI values <15% are exceptionally low for the three species, especially for *An. coluzzii* and *An. gambiae* s.s.,the most anthropophilic species of the *An. gambiae* complex. Notably, a bias due to higher numbers of non-identified human blood-meals in low quality blood-meal samples can be ruled out, as the PCR-fragment amplified for human-host is shorter than those of most other hosts^42^, and thus easier to be amplified. However, the low HBI values are parsimonious with the supposed reduced availability of human hosts to *Anopheles* vectors due to implementation of LLIN with coverage rates estimated at 90% in the study site the year prior to sampling. In fact, similar studies highlighted a plastic strategy of feeding behaviour of these usually highly anthropophilic species as a consequence of the reduced accessibility of human hosts and of the greater accessibility of readily available, although less-preferred, hosts^40,47,48^. This is consistent with the high (>70%) reported proportion of blood-meals carried out on cattle by all the 3 taxa, which is possibly associated with the presence of a settlement of pastoral Fulani families and of their cattle herds at ~400 meters from the sampled compounds.

### Sporozoite rates in *Anopheles coluzzii* sub-samples

Sporozoite rates of *An. coluzzii* (the only taxon with an adequate sample size for more detailed studies) females were higher for the human-fed sub-sample (22.9%; N = 35) than for animal-fed (8.4%; N = 143) or unfed-gravid subsamples (5.5%; N = 200), as well as for the sub-sample of fed-females with unidentified blood-meals (5.6%; N = 125) (GLMM-3; Supplementary File 3). This is in agreement with previous studies suggesting that females that have already taken a blood-meal on a (infected) human host and/or that are ready to transmit the malaria parasite, have a higher tendency to bite human again^49–51^. It is believed that *Plasmodium* can potentially affect the complex mosquito host-seeking behaviour (in the experimental model *P. yoelii*-*An. stephensi*) due to neurophysiological mechanism, based on changes in the responsiveness of mosquito odorant receptors^52^. Thus, the higher SR observed in human-fed *A. coluzzii* could be due to a significant increase in infected mosquito attraction to human odours triggered by the parasite itself in order to enhance its chances of transmission to the appropriate host^49–51^.

### Systematic review of Human Blood Index and Sporozoite Rate

The systematic review performed on published field studies indexed in PubMed reporting both SR and HBI in *An. gambiae* complex species retrieved 133 articles assessed for eligibility, of which 49 were retained according to eligibility criteria (see Materials and Methods), corresponding to 88 records that were considered in the analysis (Supplementary File 4). The proportion meta-analysis of the published data show a high inconsistency (I^2^) among studies/records in all subsamples (Table 4). Random effects estimates of HBI and SR show that the two estimates are reduced in presence of bednets when all members of the *An. gambiae* complex are combined. This pattern is confirmed also for *An. arabiensis*, while in the case of *An. coluzzii* and *An. gambiae* s.s. data were not sorted for bednet presence because of the low numbers of records, due to their recent recognition as separate species^53^. HBI values obtained from meta-analysis are far higher than the HBI recorded in the present study, even if only villages with bednets are included in the meta-analysis (*An. gambiae* s.l. 95% c.i. = 55.5–75.3%).

**Table 4:** Estimates of HBI and SR for members of the *An. gambiae* complex estimated by meta-analysis.

	**HBI**			**SR**		
	Frequency (95% C.I.)	Cochran Q (d.f.)	I^2^ (95% C.I.)	Frequency (95% C.I.)	Cochran Q (d.f.)	I^2^ (95% C.I.)
***Anopheles gambiae s.l*** *.*						
All studies	66.6%	13109.8**	99.3%	1.6%	2566.1**	96.6%
	(59.6–73.2%)	(87)	(99.3–99.4%)	(1.2–2%)	(87)	(96.3–96.9%)
Presence of bednets	65.8%	6005.3**	99.4%	0.8%	981.39**	96.4%
	(55.5–75.3%)	(35)	(99.4–99.4%)	(0.5–1.2%)	(35)	(95.9–96.8%)
No bednets	87%	919.9**	99%	5%	94.5**	90.5%
	(73.7–96%)	(9)	(98.9–99.1%)	(3.2–7.2%)	(9)	(85.1–93.3%)
***Anopheles arabiensis***						
All studies	53.8%	6849**	99.3%	0.9%	924.9**	94.7%
	(44.9–62.6%)	(49)	(99.2–99.3%)	(0.6–1.2%)	(49)	(94–95.3%)
Presence of bednets	58.5%	3817.4**	99.5%	0.5%	309**	93.5%
	(45.2–71.2%)	(20)	(99.4–99.5%)	(0.2–0.8%)	(20)	(91.8–94.7%)
No bednets	73%	541.9**	99.4%	2.3%	6.5^NS^	53.9%
	(30.7–98.8%)	(3)	(99.3–99.5%)	(1.3–3.6%)	(3)	(0–82.9%)
***Anopheles coluzzii***						
All studies	54.8%	17.8**	88.8%	0.5%	15.2*	86.9%
	(39.3–69.9%)	(2)	(58.3–94.5%)	(0–1.6%)	(2)	(42.8–93.9%)
***Anopheles gambiae s.s*** *.*						
All studies	93.4%	124.2**	95.2%	5.8%	171.5**	96.5%
	(88.2–97.1%)	(6)	(92.9–96.5%)	(2.5–10.4%)	(6)	(95.1–97.3%)

Data are based on a systematic review of 88 records of field samplings retrieved from 48 articles published between 1964 and 2016. Data are sorted for bednet presence in sampled villages. Cochran’s Q test assesses whether the proportion of observed studies is consistent. Inconsistency test (I^2^) describes the percentage of total variation across studies that is due to heterogeneity rather than chance. *P = 0.0005. **P < 0.0001.

**Figure 4:**
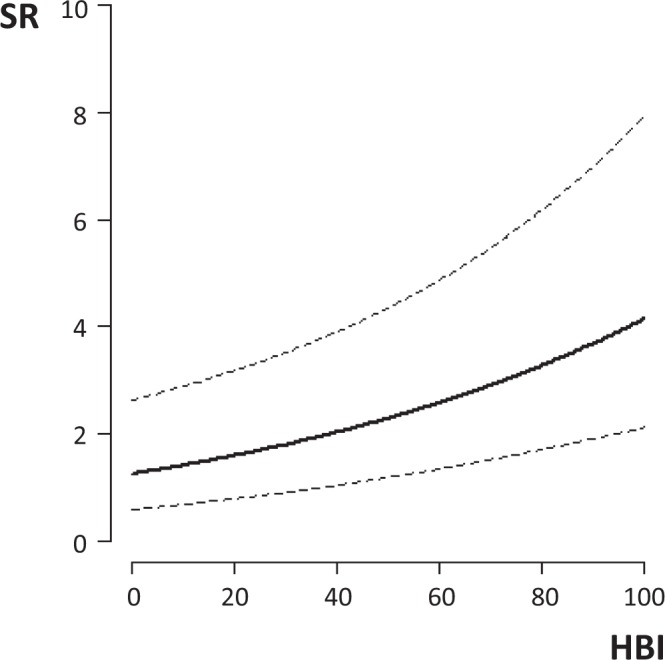
Results of Beta GLM-b investigating the relationship between Human Blood Indexes (HBI) and Sporozoite Rates (SR) calculated from data retrieved by systematic review. Solid line = fitted values; Dashed lines = 95% confidence intervals.

A positive association was shown between SR and HBI (Fig. 4, Table (a) in Supplementary File 5) also when the analysis was restricted to the 46 records including data on mosquito net use (untreated, ITN or LLIN, Table (b) and Figure (a) in Supplementary File 5). Significantly higher SRs were reported in the 10/46 records stating lack of mosquito net usage; however, this result should be interpreted with care because of the small sample size and the presence of a single record with HBI < 80% that affects the reliability of dataset for the whole range of HBI.

**Figure 5:**
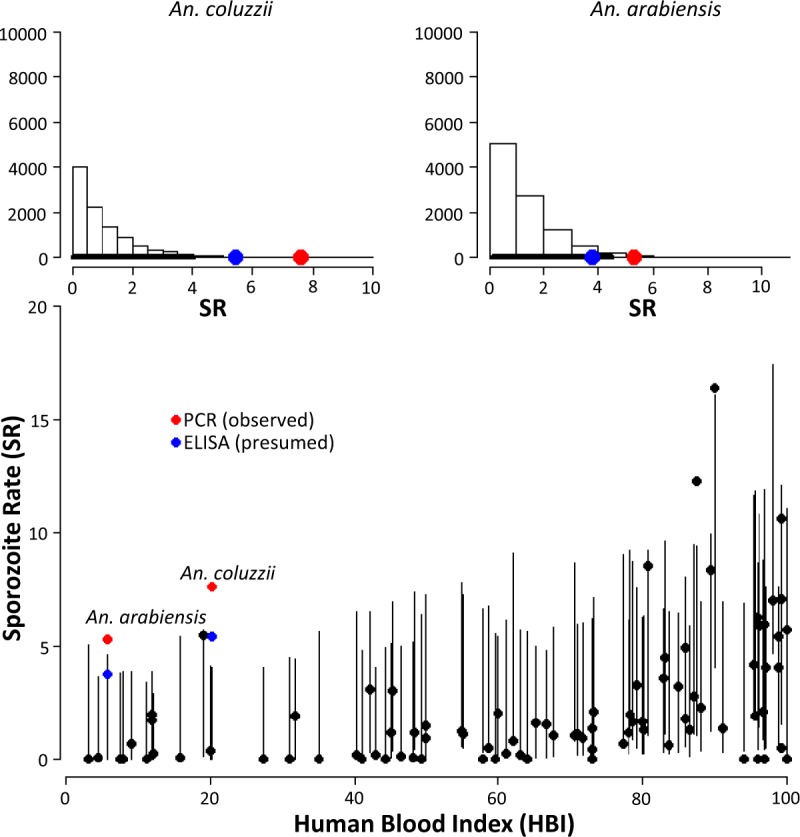
Results of the Beta GLM-b investigating the relationship between Sporozoite rates (SR) and Human-Blood Index (HBI). Upper histograms represent the distribution of SR for HBI values observed in this study; x axis represents the SR expressed in percent values; y axis represent the frequency of events occurred in the simulation (see Materials & Methods); black lines are the 95% confidence intervals (bold for meta-analysis estimate, narrow for our study). Lower plot represent the observed values of HBI and SR (black dots). Vertical bands are the 95% confidence intervals of the expected SR value from the Beta GLM. Red dots represent SR obtained by nested-PCR. Blue dots represent SR obtained by nested-PCR halved according to the higher sensitivity of nested-PCR compared to widely used ELISA method.

Beta GLM-b investigating the relationship between SR and HBI showed that the observed SRs obtained by nested-PCR in the current study largely exceeded the 95% confidence intervals for both *An. coluzzii* and *An. arabiensis* (red dots, Fig. 5; Figure (b) Supplementary File 5). This is still the case if *An. coluzzii* SRs were reduced to 5.4%, based on results from Calzetta *et al*.^54^ reporting a 1.4-fold higher sensibility of nested-PCR compared to ELISA, i.e. the most commonly used method exploited in the quoted publications) (Fig. 5; Figure (b) Supplementary File 5). However the underrepresentation of published studies reporting very low HBI may affect the reliability of the intervals for the calculated range of the corresponding SR.

It should be noted that HBI values utilized in the present study were obtained from specimens with successful blood meal origin identification. This implies that these values, as discussed above, likely represent an overestimation of actual number of blood-meals taken on humans. It is therefore overall safe to conclude that, particularly in the case of *An. coluzzii*, the SR in the study site was significantly higher than expected based on the low HBI observed and on the consequent lower probability for the mosquito to come in contact with *Plasmodium* parasite. Although less striking, a similar trend is also observed for *An. arabiensis*.

## Conclusions

LLINs provide protection from malaria to net users by both the physical barrier of the net, which reduces contact with the mosquito, and by the insecticide within the net fibres, which reduces the likelihood that a mosquito will feed through the net. In addition, they provide protection to non-users by reducing the total mosquito population and shortening their life span. This community effect is observed when net usage rates exceed 50% in the population^55^. In the Burkinabe village of Goden, LLINs had been distributed the year prior to the study and reported net usage was highly above this value. Thus, one would have expected human vector-contact and sporozoite rates to be reduced in people both protected and unprotected by bednets, also considering the presence of alternative hosts (cattle) in the village^55^. Instead, the main malaria vector species in Goden showed low HBI values and rates of *Plasmodium* infection in the range of those normally observed in the presence of much higher rates of feeding on humans typical of these species in absence of extensive LLIN coverage. It can be hypothesized that, even if HBI is reduced due to mosquitoes feeding on alternative hosts, as humans are largely protected by LLINs, the infectious reservoir could be being maintained by non-bednet users if the local vector population was resilient to the toxic effects of the pyrethroids on the nets. Although we did not measure insecticide resistance in Goden and no data are available in literature for this village, pyrethroid resistance is widespread in Burkina Faso^25,56–59^ and may have played a role by reducing daily mosquito mortality, lengthening the vector population average age and increasing the risk of malaria transmission to people when unprotected by a bednet^37^.

In the study site the fraction of the human population not protected by a bednet and thus more readily available to the vectors may thus constitutes a “core group” of people who are repeatedly exposed to mosquito bites. These people would represent the major reservoir of *Plasmodium* gametocytes capable of maintaining the high levels of infection observed in the vector population. These high levels of mosquito infection, in association with high mosquito densities^32,60^ may sustain the transmission even to the fraction of the inhabitants commonly protected by a bednet. Although we cannot exclude that some people from Goden have been infected by travelling in other areas, the above hypothesis is supported by modelling studies showing that the effect of uneven distribution of mosquito bites - with a “core group” of people more exposed to infections than the rest of the population - is associated with an increase in human-to-mosquito transmission efficiency as malaria is controlled^61^. If confirmed by additional studies, this would highlight the need to specifically target the “core group” of people sustaining the transmission in order to achieve a significant reduction in transmission.

Despite the limitations of this study (i.e. lack of SR and HBI data before large-scale LLIN distribution and lack of clinical data on malaria incidence), these results may contribute to explain the low effect of LLIN distribution on malaria incidence in some African regions, such as Burkina Faso. Further studies measuring HBI and sporozoite rates in areas of high levels of pyrethroid resistance, accompanied by information on net usage and the prevalence of malaria in bednet users versus non-bednet users, are needed to establish whether the pattern we report in this study reflects contemporary patterns of malaria transmission in areas with high mosquito densities and high (but not complete) LLIN use.

## Methods

### Study site

Field collections were carried out from August to November 2011 in the village of Goden (Ziniaré health district; 12°25′N–1°21′W) located in a Sudanese-savanna area 41 km East of Ouagadougou, the capital city of Burkina Faso. Goden is a rural village with approximately 800 inhabitants mainly belonging to the Mossi ethnic group, mostly devoted to agriculture and rearing a few animals (e.g. pigs, dogs, goats and chickens) in their compounds. In the year of sampling, two families belonging to Fulani nomadic pastoral ethnic group settled nearby the village with approximately 200 bovines, representing the second most abundant host for mosquitoes. LLINs were distributed in 2010 to approximately 90% of the population (N’F S and MG, unofficial data).

### Mosquito sampling and species identification

Field sampling is described in Pombi *et al*.^32^. In brief, mosquitoes were collected from 5 compounds, 2 houses per compound (10 in total), randomly chosen in the village, where LLINs were available. The sampling has been done in the same houses for the whole sampling season. Indoor and outdoor resting *Anopheles* mosquitoes were collected by back-pack aspirations either inside houses (BP) or in pit-shelters (PIT) and by sticky resting boxes (SRB). Mosquitoes glued on sticky sheets were removed by cutting out a small sheet fragment around them and washing it with acetone for 2 minutes. All collected mosquitoes were morphologically identified under a stereomicroscope^62^, separated by species, gender and gonotrophic stage (i.e. unfed, freshly/fully fed, half-gravid, gravid) and stored in individual tubes containing silica-gel. DNA was extracted from entire specimens in the case of SRB collections, and from heads + thoraxes and abdomens separately in the case of BP and PIT. *Anopheles gambiae* species complex were identified by PCR^32^.

### Plasmodium detection and blood meal identification

DNA extracted from either heads + thoraxes or abdomens in the case of BP- and PIT-collected specimens (in order to distinguish between infective and infected mosquitoes), and from entire specimens in the case of SRB collections (which could not be easily dissected due to the glue presence) were processed by nested-PCR protocol targeting mtDNA to detect *Plasmodium* sp.^54^. A subsample of PCR products from *Plasmodium* positive specimens was sequenced (BMR genomics sequencing service, Padua, Italy) for species identification by BLAST (National Centre for Biotechnology Information, Bethesda, MD, USA).

DNA from either abdomens (BP and PIT) or entire specimens (SRB) was used as template for multiplex-PCR protocol targeting mtDNA to detect blood meal origin^42^; primers for human, cow, pig, dog and goat were used.

### Statistical analyses

Generalized Linear Mixed Models (GLMM) and Generalized Linear Model (GLM) were performed assuming a binomial distribution with logit link. To incorporate a dependency structure among observations from the same house or in the same day the variable house was considered as a random effect nested into the variable compound, while the variable collection days/weeks was considered as a crossed random effect. Assessment of model assumption was carried out by graphically inspect Pearson residuals versus fitted values and each covariate.

GLMM-1 was carried out to investigate whether the probability of a mosquito being infected by *Plasmodium* sp. is different in *Anopheles gambiae* complex species *(An. arabiensis, An. coluzzii, An. gambiae* s.s.) accounting for collection periods (i.e. month) and sampling positions (indoors or outdoors). As part of the model validation process, 10,000 data sets have been simulated from the model to investigate if the predicted probability of infected mosquito was in accordance with the observed value (see Supplementary File 1).

The following analyses were carried out on *Anopheles coluzzii* and *An. arabiensis* (i.e. the two most represented taxa) from BP and PIT collections. *Plasmodium* infection in abdomen and in head-thorax was regressed against the taxa by means of Binomial GLMM.

GLM-a was carried assuming a binomial distribution with logit link out to assess differences in blood meal detection between sticky (SRB) and non-sticky methods (BP + PIT), as well as in fed mosquitoes with different degrees of digestion of blood for *An. coluzzii* fed mosquitoes. Tukey post-hoc test was carried out to assess the significance of all pairwise comparison. A simulation study was carried out to understand how the different sample size in each group affected the results (see Supplementary File 2).

GLMM-2 was carried out to investigate whether the probability of mosquito having fed on human was different in *Anopheles gambiae* complex species *(An. arabiensis, An. coluzzii*) accounting for indoors or outdoors collections. The model was fitted only on the subsample of identified blood meal (see Table (b) on Supplementary File 2).

GLMM-3 was carried out to investigate whether the source of blood meal (blood from human, animal or unidentified hosts plus unfed/gravid mosquitoes) affected the probability of positive infection to sporozoite in specimens of *An. coluzzii*.

### Systematic review and meta-analysis

A systematic search and review of field studies indexed in the publication repository PubMed (www.ncbi.nlm.nih.gov/pubmed) was carried out for studies published in English, French or Italian between 1964 and August 2016. As no review protocol are already available for this systematic review, articles were searched using the keywords “gambiae”, “coluzzii”, and “arabiensis” in association with any one of the following keywords: “human blood index” (or “HBI”), “blood meal sporozoite”, “blood index Plasmodium”, “human blood sporozoite index”. From the initial set of publications the following information were retrieved and recorded in an Excel database: country of collection, village/area of collection, sampling season (dry-rainy), year of collection, presence of bednets (untreated, ITN, LLIN), mosquito species collected, human blood index, HBI sample size, protocol for HBI detection (ELISA, PCR, other), sporozoite rate (SR), SR sample size, protocol for SR detection (ELISA, PCR, other) whenever available. Articles not reporting both HBI and SR with their relative sample size were excluded from the analysis. A meta-analysis was performed to obtain weighted estimates of mean values of SR and HBI. Homogeneity across studies was assessed by the Cochran Q and I^2^ statistics^63,64^. Whenever the P-value of Cochran Q was greater than 5% and it was associated with a value of I^2^ ≤ 25%, the studies were considered homogeneous and the SR and HBI were calculated considering a fixed-effects combined value. Conversely, if the studies were significantly heterogeneous, a random-effects combined value was calculated. Under the fixed-effect model it is assumed that the true effect is the same in all studies, while under the random-effects model allowance is made for the true effect to vary across studies^65^.

The relationship between HBI and SR values obtained from the systematic review of the literature was investigated by means of GLM-b assuming a beta distribution with logit link. Since SR values included zeroes, which are excluded by the beta distribution, the transformation (Y*(N-1) + 0.5)/N was employed^66^. Y is the SR values and N the sample size. Transformed SR values were regressed against HBI values accounting for sampling season, region and species studied. Information on the presence of simple Mosquito Nets and Insecticide Treated Nets (including LLINs) was not available for all the studies considered. Therefore, to investigate the effects of mosquito nets on SR, Beta GLM-c was carried out using only the subsample with available data. HBI and Presence/Absence of Mosquito Nets were considered as independent variables. Finally, the expected SR values for the data observed in this study was computed using both Beta GLMs and compared to the actual value observed.

Statistical analysis were carried out using Stats Direct statistical software^67^, R statistical software^64^ version 3.3.2 and the lme4^68^, betareg^69^, plyr^70^ packages.

## Electronic supplementary material

Supplementary Information
