# Supplementary material for: Unexpectedly high Plasmodium sporozoite rate associated with low human blood index in Anopheles coluzzii from a LLIN-protected village in Burkina Faso
Source: Sci Rep. 2018 Aug 24;8:12806. doi: 10.1038/s41598-018-31117-x (PMC6109043; doi:10.1038/s41598-018-31117-x)
Supplement: Supplementary file 1 — Supplementary Information [file 41598_2018_31117_MOESM1_ESM.docx]

**Unexpectedly high *Plasmodium* sporozoite rate associated with low human blood index in *Anopheles coluzzii* from a LLIN-protected village in Burkina Faso.**

Marco Pombi^1,^*, Maria Calzetta^1^, Wamdaogo M. Guelbeogo^2^, Mattia Manica^1,3^, Eleonora Perugini^1^, Verena Pichler^1^, Emiliano Mancini^4^, N’Fale Sagnon^2^, Hilary Ranson^5^, and Alessandra della Torre^1^

^1^ Dipartimento di Sanità Pubblica e Malattie Infettive, Laboratory affiliated to Istituto Pasteur Italia - Fondazione Cenci Bolognetti, Sapienza Università di Roma, Rome, 00185, Italy

^2^ Centre National de Recherche et Formation sur le Paludisme (CNRFP), Ouagadougou, 01 BP 2208, Burkina Faso

^3^ Dipartimento di Biodiversità ed Ecologia Molecolare, Centro Ricerca e Innovazione, Fondazione Edmund Mach, via E. Mach 1, 38010 San Michele all'Adige, Italy
^4^ Università di “Roma Tre”, Dipartimento di Scienze, Rome, 00154, Italy
^5^ Liverpool School of Tropical Medicine, Department of Vector Biology, Liverpool, L3 5QA, UK

* Correspondence to: marco.pombi@uniroma1.it

**SUPPLEMENTARY MATERIAL**

**Supplementary File 1.**

|  | **Estimate** | **Std. Error** | **z value** | **P-value** |
| --- | --- | --- | --- | --- |
| Intercept | -2.09473 | 0.34123 | -6.139 | *<0.0001* |
| taxonM | 0.19364 | 0.24085 | 0.804 | 0.421 |
| taxonS | 0.36949 | 0.52100 | 0.709 | 0.478 |
| November | 0.04890 | 0.32676 | 0.150 | 0.881 |
| October | -0.01264 | 0.33733 | -0.037 | 0.970 |
| September | -0.40663 | 0.34973 | -1.163 | 0.245 |
| Pit-ShelterOUT | 0.04511 | 0.25474 | 0.177 | 0.859 |
| SRBIN | -0.07546 | 0.36011 | -0.210 | 0.834 |
| SRBOUT | -0.34150 | 0.27866 | -1.226 | 0.220 |

**Table a.** Estimated regression parameters, standard errors, z-values and P-values for the Binomial GLMM-1 investigating presence of *Plasmodium*. The estimated value for the random effect Compound, House, Week are: 6*10^-5^. 2*10^-5^. 0.17

**Whole Mosquito Infection Simulation study**

**Methods**: see main text for details


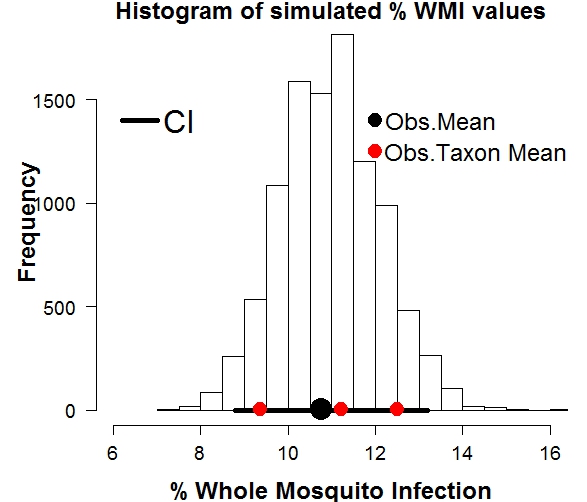


**Figure a.** Histogram of the frequency of whole mosquito infection (WMI) proportion in the10,000 simulated data sets from the Binomial GLMM-1. The dots represents observed data: red ones the WMI proportion in the three species, the black one the overall WMI proportion.

**Supplementary File 2.**

**Blood meal simulation study**

**Methods**: in the simulation study, binomial GLMs were carried out on 1,000 datasets generated for each comparison. *i)* At first, the probability of observing a statistically significant difference (as in the result) between partially fed mosquito collected using non-sticky *vs* sticky traps was tested. The probabilities of blood meal detection were equally fixed for both groups at the value estimated by GLM-a for the partially fed mosquito collected using non-sticky traps. The sample sizes were kept as of the original database. *ii)* the probability of observing a statistically significant difference (as in the result) between fully fed mosquito collected using non-sticky *vs* sticky traps was tested. The probabilities of blood meal detection were kept as of the original database for both groups at the value estimated by GLM-a. The sample sizes were kept as of the original database. *iii*) the probability of observing a statistically significant difference (as in the result) between fully fed *vs* partially fed mosquito collected using non-sticky traps was tested. The probabilities of blood meal detection were equally fixed for both groups at the value estimated by GLM-a for the partially fed mosquito collected using non-sticky traps. The sample sizes were kept as of the original database

The probability of erroneously detecting a significant difference between partially fed *vs* fully fed mosquito collected using non-sticky traps was computed. A binomial GLM testing partially fed *vs* fully fed differences in PCR blood meal detection was carried out on 1,000 datasets generated using for all classes the sample sizes of the original database and a binomial distribution with the same success probability equal to the blood meal detection rate of partially fed mosquitoes collected using non-sticky traps. Then, the probability of erroneously not detecting a significant difference among groups of partially *vs* fully fed mosquitoes collected using sticky traps was computed. A binomial GLM testing differences in PCR blood meal detection of groups of fed mosquitoes was carried out on 1,000 datasets generated using for all classes the sample sizes of the original database and a binomial distribution. For fully fed mosquito collected using sticky traps, the estimated blood meal detection probability was used in the generating binomial distribution. On the other hand, for partially fed mosquitoes collected using sticky traps, the estimated blood meal detection probability of those collected using non-sticky traps was used in the generating binomial distribution.

**Results**: The simulation study based on groups reported in table (a) showed that

1. the probability of erroneously detecting a significant difference (p-value < 0.05) between non-sticky *vs* sticky collected mosquito (partially fed) was 6.6% (although unlikely the observed difference may be not true)
2. the probability of detecting a significant difference (p-value < 0.05) between non-sticky *vs* sticky collected mosquito (fully fed) was 4.6% (so it is unlikely that there is a difference)
3. the probability of detecting a significant difference (p-value < 0.05) between partially fed *vs* fully fed mosquito (non-sticky collection) was 4.4% (so it is likely that the observed difference is “true”)
4. the probability of detecting a significant difference (p-value < 0.05) between partially fed *vs* fully fed mosquito (sticky collection) was 10.9% (so it is likely that the observed difference is “true”)
5. the probability of erroneously detecting a significant difference between partially fed (N = 241, simulated PCR blood meal detection = 0.55) *vs* fully fed mosquito (N = 57, simulated PCR blood meal detection = 0.55) collected using non-sticky traps was 0.046, *ii)* the probability of erroneously not detecting a significant difference between partially fed (N = 41, simulated PCR blood meal detection = 0.55) *vs* fully fed mosquito (N = 45, simulated PCR blood meal detection = 0.73) collected using sticky traps was 0.567.

|  | Non-Sticky | Sticky |
| --- | --- | --- |
| Fully-Fed | 57 | 45 |
| Half-Gravid | 5 | 30 |
| Partially Fed | 241 | 41 |

**Table a**. Sample size for PCR detection of blood meal

|  | **Estimate** | **Std. Error** | **z value** | **P-value** | **PCR detection rate (CI)** |
| --- | --- | --- | --- | --- | --- |
| Non-Sticky & Partially Fed | 0.1915 | 0.1294 | 1.479 | 0.13906 | 55 % (48 – 61) |
| Sticky & Partially Fed | 1.2256 | 0.4148 | 2.955 | *0.00313* | 80 % (66 – 90) |
| Non-Sticky & Half-Gravid | 0.2140 | 0.9220 | 0.232 | 0.81645 | 60 % (20 – 90) |
| Sticky & Half-Gravid | -1.0845 | 1.0719 | -1.012 | 0.31163 | 63 % (45 – 78) |
| Non-Sticky & Fully-Fed | 0.9307 | 0.3338 | 2.788 | *0.00530* | 75 % (63 – 85) |
| Sticky & Fully-Fed | -1.3362 | 0.6167 | -2.166 | *0.03028* | 73 % (59 -84) |

**Table b.** Estimated regression parameters, standard errors, z-values and P-values for the Binomial GLM-a investigating the PCR detection of blood meal in *An. coluzzii* fed mosquitoes. Estimated PCR detection rate for each class


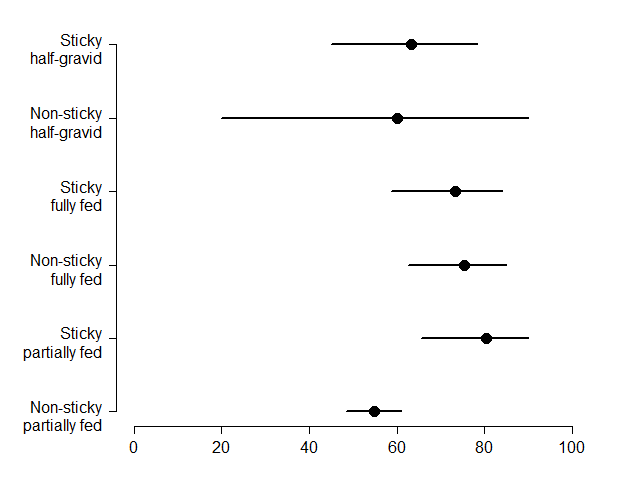


**Figure a.** Results of the Binomial GLM-a investigating the percentage of PCR detection of blood meal in *An. coluzzii* fed mosquitoes. The dots represent fitted values: horizontal bands are the 95% confidence intervals.


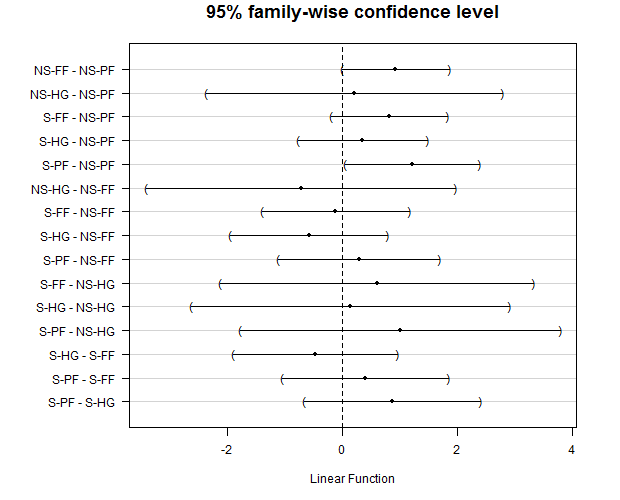


**Figure b.** Pair comparisons of the percentage of PCR detection of blood meal in *An. coluzzii* fed mosquitoes. The dots represent fitted values: horizontal bands are the 95% confidence intervals. Bars not overlapping 0-value mean significant difference between pairs. NS= non-sticky method; S= sticky methods; FF= fully-fed; PF= partially-fed; HG= half-gravid.

|  | Estimate | Std. Error | z value | P-value |
| --- | --- | --- | --- | --- |
| Intercept | -2.894 | 0.898 | -3.224 | *0.0012* |
| taxonM | 1.495 | 0.510 | 2.929 | *0.0034* |
| Outside | -1.145 | 0.428 | -2.675 | *0.0075* |

**Table c**. Estimated regression parameters, standard errors, z-values and P-values for the Binomial GLMM-2 investigating Human Blood Index. The estimated values for the random effects Compound, House, Week are: 1.37, 0.38, and 0.79

**Supplementary File 3**

|  | **Estimate** | **Std. Error** | **z value** | **P-value** |
| --- | --- | --- | --- | --- |
| Unfed/Gravid | -2.972 | 0.382 | -7.780 | *<0.0001* |
| Animal Fed | 0.472 | 0.435 | 1.086 | 0.277 |
| Human Fed | 1.743 | 0.549 | 3.177 | *0.001* |
| Unidentified | 0.036 | 0.503 | 0.072 | 0.942 |

**Table a.** Estimated regression parameters, standard errors, z-values and P-values for the Binomial GLMM-3 investigating sporozoite index. The estimated value for the random effects Compound, House, Week are: 1.6*10^-5^. 0.41. 0.21

**Supplementary File 4**

Flow diagram of the selection of records retrieved from systematic review and eligible publications used for subsequent analyses according to PRISMA statement (Moher D, Liberati A, Tetzlaff J, Altman DG, The PRISMA Group (2009) Preferred Reporting Items for Systematic Reviews and Meta-Analyses: The PRISMA Statement. PLoS Med 6(7): e1000097. See Materials and Methods for further details).


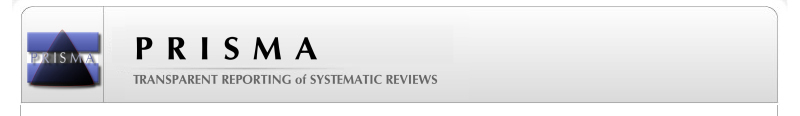
**PRISMA 2009 Flow Diagram**

Studies included in quantitative synthesis (meta-analysis)
(n = 49)

Records excluded
(n = 0)

Records screened
(n = 133)

Records after duplicates removed
(n = 133)

## Identification

## Eligibility

## Included

## Screening

Records identified through PubMed database searching (1964-Aug 2016)
(n = 264)

Additional records identified through other sources
(n = 4)

Full-text articles excluded, with reasons
(n = 84)

Full-text articles assessed for eligibility
(n = 133)

Studies included in qualitative synthesis
(n = 49)

List of articles included in the systematic review and meta-analysis for the estimates of Human Blood Indexes and Sporozoite Rates in *Anopheles gambiae* complex species.

1. Abdalla, H. *et al.* Insecticide susceptibility and vector status of natural populations of Anopheles arabiensis from Sudan. *Trans. R. Soc. Trop. Med. Hyg.* **102,** 263–271 (2008).
2. Animut, A., Balkew, M., Gebre-Michael, T. & Lindtjørn, B. Blood meal sources and entomological inoculation rates of anophelines along a highland altitudinal transect in south-central Ethiopia. *Malar. J.* **12,** 76 (2013).
3. Bogh, C., Clarke, S. E., Pinder, M., Sanyang, F. & Lindsay, S. W. Effect of passive zooprophylaxis on malaria transmission in the Gambia. *J. Med. Entomol.* **38,** 822–828 (2001).
4. Calzetta, M. *et al.* Distribution and chromosomal characterization of the Anopheles gambiae complex in Angola. *Am. J. Trop. Med. Hyg.* **78,** 169–75 (2008).
5. Charlwood, J. D. *et al.* The impact of indoor residual spraying with Malathion on malaria in refugee camps in eastern Sudan. *Acta Trop.* **80,** 1–8 (2001).
6. Chirebvu, E. & Chimbari, M. J. Characterization of an Indoor-Resting Population of Anopheles arabiensis (Diptera: Culicidae) and the Implications on Malaria Transmission in Tubu Village in Okavango Subdistrict, Botswana. *J. Med. Entomol.* **53,** 569–576 (2016).
7. Fontenille, D. *et al.* MALARIA TRANSMISSION AND VECTOR BIOLOGY IN MANARINTSOA, HIGH PLATEAUX OF MADAGASCAR. *Am. J. Trop. Med. Hyg.* **43,** 107–115 (1990).
8. Fornadel, C. M. & Norris, D. E. Increased endophily by the malaria vector Anopheles arabiensis in southern Zambia and identification of digested blood meals. *Am. J. Trop. Med. Hyg.* **79,** 876–880 (2008).
9. Fornadel, C. M., Norris, L. C., Glass, G. E. & Norris, D. E. Analysis of Anopheles arabiensis blood feeding behavior in southern zambia during the two years after introduction of insecticide-treated bed nets. *Am. J. Trop. Med. Hyg.* **83,** 848–853 (2010).
10. Habtewold, T., Walker, A. R., Curtis, C. F., Osir, E. O. & Thapa, N. The feeding behaviour and Plasmodium infection of Anopheles mosquitoes in southern Ethiopia in relation to use of insecticide-treated livestock for malaria control. *Trans. R. Soc. Trop. Med. Hyg.* **95,** 584–586 (2001).
11. Himeidan, Y. E., Elzaki, M. M., Kweka, E. J., Ibrahim, M. & Elhassan, I. M. Pattern of malaria transmission along the Rahad River basin, Eastern Sudan. *Parasit. Vectors* **4,** 109 (2011).
12. Ijumba, J. N., Mosha, F. W. & Lindsay, S. W. Malaria transmission risk variations derived from different agricultural practices in an irrigated area of northern Tanzania. *Med Vet Entomol* **16,** 28–38 (2002).
13. Jawara, M. *et al.* Dry season ecology of Anopheles gambiae complex mosquitoes in The Gambia. *Malar. J.* **7,** 156 (2008).
14. Kabula, B. *et al.* A significant association between deltamethrin resistance, Plasmodium falciparum infection and the Vgsc-1014S resistance mutation in Anopheles gambiae highlights the epidemiological importance of resistance markers. *Malar. J.* **15,** 289 (2016).
15. Kasili, S. *et al.* Entomological assessment of the potential for malaria transmission in Kibera slum of Nairobi, Kenya. *J. Vector Borne Dis.* **46,** 273–279 (2009).
16. Kerah-Hinzoumbé, C. *et al.* Malaria vectors and transmission dynamics in Goulmoun, a rural city in south-western Chad. *BMC Infect. Dis.* **9,** 71 (2009).
17. Kibret, S. *et al.* The impact of a small-scale irrigation scheme on malaria transmission in Ziway area, Central Ethiopia. *Trop. Med. Int. Heal.* **15,** 41–50 (2010).
18. Kibret, S., Lautze, J., Boelee, E. & McCartney, M. How does an Ethiopian dam increase malaria? Entomological determinants around the Koka reservoir. *Trop. Med. Int. Heal.* **17,** 1320–1328 (2012).
19. Kibret, S., Wilson, G. G., Tekie, H. & Petros, B. Increased malaria transmission around irrigation schemes in Ethiopia and the potential of canal water management for malaria vector control. *Malar. J.* **13,** 1–12 (2014).
20. Kipyab, P. C., Khaemba, B. M., Mwangangi, J. M. & Mbogo, C. M. The bionomics of Anopheles merus (Diptera: Culicidae) along the Kenyan coast. *Parasit. Vectors* **6,** 1 (2013).
21. Lefèvre, T. *et al.* Beyond nature and nurture: phenotypic plasticity in blood-feeding behavior of Anopheles gambiae s.s. when humans are not readily accessible. *Am. J. Trop. Med. Hyg.* **81,** 1023–9 (2009).
22. Lemasson, J. J. *et al.* Comparison of behavior and vector efficiency of Anopheles gambiae and An. arabiensis (Diptera:Culicidae) in Barkedji, a Sahelian area of Senegal. *J. Med. Entomol.* **34,** 396–403 (1997).
23. Lindsay, S. W. *et al.* Ability of anopheles-gambiae mosquitos to transmit malaria during the dry and wet seasons in an area of irrigated rice cultivation in the gambia. *J. Trop. Med. Hyg.* **94,** 313–324 (1991).
24. Magbity, E. B. *et al.* Effects of community-wide use of lambdacyhalothrin-impregnated bednets on malaria vectors in rural Sierra Leone. *Med. Vet. Entomol.* **11,** 79–86 (1997).
25. Mala, A. O. *et al.* Plasmodium falciparum transmission and aridity: a Kenyan experience from the dry lands of Baringo and its implications for Anopheles arabiensis control. *Malar. J.* **10,** 121 (2011).
26. Mayagaya, V. S. *et al.* The impact of livestock on the abundance, resting behaviour and sporozoite rate of malaria vectors in southern Tanzania. *Malar. J.* **14,** 17 (2015).
27. Mbogo, C. N., Baya, N. M., Ofulla, A. V, Githure, J. I. & Snow, R. W. The impact of permethrin-impregnated bednets on malaria vectors of the Kenyan coast. *Med. Vet. Entomol.* **10,** 251–9 (1996).
28. Merzagora, L. Variazioni ecologiche ed epidemiologia della malaria in una zona di savana sudanese presso Ouagadougou Burkina Faso. (Università degli Studi di Roma ‘La Sapienza’, 1993).
29. Mint Lekweiry, K. *et al.* Circumsporozoite protein rates, blood-feeding pattern and frequency of knockdown resistance mutations in Anopheles spp. in two ecological zones of Mauritania. *Parasit. Vectors* **9,** 268 (2016).
30. Muturi, E. J. *et al.* Effect of rice cultivation on malaria transmission in central Kenya. *Am. J. Trop. Med. Hyg.* **78,** 270–275 (2008).
31. Mwangangi, J. M. *et al.* Blood-meal analysis for anopheline mosquitoes sampled along the Kenyan coast. *J Am Mosq Control Assoc* **19,** 371–375 (2003).
32. Mzilahowa, T., Hastings, I. M., Molyneux, M. E. & McCall, P. J. Entomological indices of malaria transmission in Chikhwawa district, Southern Malawi. *Malar. J.* **11,** 380 (2012).
33. Obala, A. A. *et al.* Anopheles gambiae and Anopheles arabiensis population densities and infectivity in Kopere village, Western Kenya. *J. Infect. Dev. Ctries.* **6,** 637–643 (2012).
34. Petrarca, V. *et al.* Species composition of the Anopheles gambiae complex (Diptera: Culicidae) at two sites in western Kenya. *J. Med. Entomol.* **28,** 307–313 (1991).
35. Petrarca, V., Vercruysse, J. & Coluzzi, M. Observations on the Anopheles gambiae complex in the Senegal River Basin, West Africa. *Med. Vet. Entomol.* **1,** 303–12 (1987).
36. Rajaonarivelo, V., Le Goff, G., Cot, M. & Brutus, L. Les anophèles et la transmission du paludisme à Ambohimena, village de la marge occidentale des Hautes-Terres malgaches. *Parasite* **11,** 75–82 (2004).
37. Robert, V. *et al.* Detection of falciparum malarial forms in naturally infected anophelines in Cameroon using a fluorescent anti-25-kD monoclonal antibody. *Am. J. Trop. Med. Hyg.* **52,** 366–369 (1995).
38. Robert, V. *et al.* Mosquitoes and malaria transmission in irrigated in the Benoue valley of northern Cameroon. *Acta Trop.* **52,** 201–204 (1992).
39. Robert, V. *et al.* Moderate transmission but high prevalence of malaria in Madagascar. *Int. J. Parasitol.* **36,** 1273–1281 (2006).
40. Shililu, J. *et al.* Seasonal abundance, vector behavior, and malaria parasite transmission in Eritrea. *J. Am. Mosq. Control Assoc.* **20,** 155–64 (2004).
41. Tanga, M. C., Ngundu, W. I. & Tchouassi, P. D. Daily survival and human blood index of major malaria vectors associated with oil palm cultivation in Cameroon and their role in malaria transmission. *Trop. Med. Int. Health* **16,** 447–457 (2011).
42. Tchouassi, D. P. *et al.* Characterization of malaria transmission by vector populations for improved interventions during the dry season in the Kpone-on-Sea area of coastal Ghana. *Parasit. Vectors* **5,** 212 (2012).
43. Tchuinkam, T. *et al.* Bionomics of Anopheline species and malaria transmission dynamics along an altitudinal transect in Western Cameroon. *BMC Infect. Dis.* **10,** 119 (2010).
44. Tirados, I., Costantini, C., Gibson, G. & Torr, S. J. Blood-feeding behaviour of the malarial mosquito Anopheles arabiensis: Implications for vector control. *Med. Vet. Entomol.* **20,** 425–437 (2006).
45. Waka, M., Hopkins, R. J., Akinpelu, O. & Curtis, C. Transmission of malaria in the Tesseney area of Eritrea: parasite prevalence in children, and vector density, host preferences, and sporozoite rate. *J. Vector Ecol.* **30,** 27–32 (2005).
46. Wamae, P. M., Githeko, A. K., Otieno, G. O., Kabiru, E. W. & Duombia, S. O. Early biting of the Anopheles gambiae s.s. and its challenges to vector control using insecticide treated nets in western Kenya highlands. *Acta Trop.* **150,** 136–142 (2015).
47. Wanji, S. *et al.* Anopheles species of the mount Cameroon region: Biting habits, feeding behaviour and entomological inoculation rates. *Trop. Med. Int. Heal.* **8,** 643–649 (2003).
48. White, G. B. & Rosen, P. Comparative studies on sibling species of the Anopheles gambiae Giles complex (Dipt., Culicidae). II. Ecology of species A and B in savanna around Kaduna, Nigeria,. *Bull. Entomol. Res.* **62,** 613–615 (1973).
49. Yohannes, M. *et al.* Can source reduction of mosquito larval habitat reduce malaria transmission in Tigray, Ethiopia? *Trop. Med. Int. Heal.* **10,** 1274–1285 (2005).

**Supplementary File 5**

|  | **Estimate** | **Std. Error** | **z value** | **P-value** |
| --- | --- | --- | --- | --- |
| Intercept | -4.37278 | 0.38749 | -11.285 | *<0.0001* |
| HBI | 1.23324 | 0.29136 | 4.233 | *<0.0001* |
| Region E | -0.06706 | 0.28072 | -0.239 | 0.811192 |
| Region S | -0.17893 | 0.85801 | -0.209 | 0.834805 |
| Region W | -0.26146 | 0.22650 | -1.154 | 0.248361 |
| Dry/Rainy | -0.31293 | 0.23587 | -1.327 | 0.184613 |
| Rainy | 0.24222 | 0.23872 | 1.015 | 0.310276 |
| *An. coluzzii* | 0.41478 | 0.50338 | -0.824 | 0.409948 |
| *An. gambiae s.l.* | 0.26466 | 0.27780 | 0.953 | 0.340746 |
| *An. gambiae s.s.* | 0.80491 | 0.20946 | 3.843 | *0.000122* |
| *An. merus* | -0.60222 | 0.57693 | -1.044 | 0.296560 |
| Phi coefficient | 85 | 13.52 | 6.288 | <0.0001 |

**Table a.** Estimated regression parameters, standard errors, z-values and P-values for the Beta GLM-b investigating the relationship between HBI and SR. Sample size N = 88

|  | **Estimate** | **Std. Error** | **z value** | **P-value** |
| --- | --- | --- | --- | --- |
| Intercept | -4.2508 | 0.2503 | -16.982 | *<0.0001* |
| HBI | 0.8784 | 0.3286 | 2.673 | *0.00751* |
| No Nets | 0.7098 | 0.1749 | 4.059 | *<0.0001* |
| Phi coefficient | 99.55 | 21.40 | 4.652 | *<0.0001* |

**Table b.** Estimated regression parameters, standard errors, z-values and P-values for the Beta GLM-c investigating the effect of mosquito nets on the relationship between HBI and SR. Sample size N = 46

**
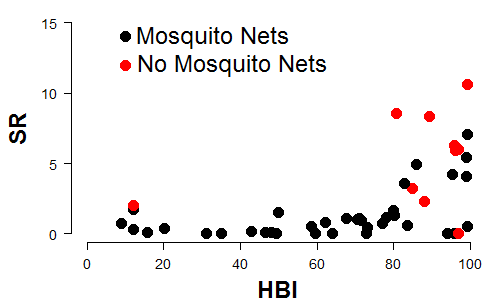
**

**Figure a.** Observed values for HBI (x-axis) and SR (y-axis) of studies having information about the use of mosquito nets


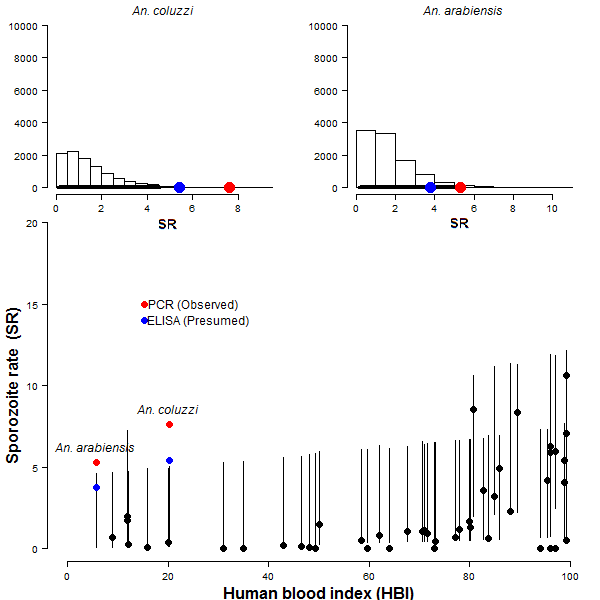


**Figure b.** **Results of the Beta GLM-c investigating the relationship between HBI and SR in published studies reporting data on villages where bednets were present.** Upper histograms represent the distribution of SRs for HBI values observed in this study; x axis represents the SR expressed in percent values; y axis represent the frequency of events occurred in the simulation (see Materials & methods); black lines are the 95% confidence intervals (bold for meta-analysis estimate, narrow for our study). Lower plot represent the observed values of HBI and SR (black dots). The vertical bands represent the 95% confidence intervals of the expected SR value from the Beta GLM. Red dots are the SR value observed in this study, blue dots are presumed values of SR reduced according to the higher sensitivity of nested-PCR method used compared to ELISA. On the y-axis the SR, on the x-axis the HBI.
